# Profile of regional fat and fat-free soft tissue accumulation in male athletes

**DOI:** 10.1186/s40101-020-0215-0

**Published:** 2020-03-06

**Authors:** Yohei Takai, Miyuki Nakatani, Toru Aoki, Daisuke Komori, Kazuyuki Oyamada, Kensuke Murata, Eiji Fujita, Takuya Akamine, Yoshihisa Urita, Masayoshi Yamamoto, Hiroaki Kanehisa

**Affiliations:** 1 National Institute of Fitness and Sports in Kanoya, 1 Shiromizu, Kanoya, Kagoshima, 891-2393 Japan; 2 Faculty of Sport and Health Science, Ritsumeikan University, 1-1-1 Noji-higashi, Kusatsu, Shiga 525-8577 Japan

**Keywords:** Body composition, Piecewise regression analysis, Breakpoint, Regional difference

## Abstract

**Background:**

It is unclear whether or not the breakpoint (BP), at which the proportion of each of fat mass (FM) and fat-free soft tissue mass (FFSTM) to body mass (BM) alter, exists in male athletes. We examined the hypothesis that in male athletes, the regional FM and FFSTM-BM relationships have a BP, but the body mass at BP (BM_BP_) differs among the arms, trunk, and legs.

**Methods:**

By using a dual X-ray absorptiometry, whole-body and regional FMs and FFSTMs in the arms, trunk, and legs were estimated in 198 male athletes (20.8 ± 2.1 years; 1.73 ± 0.07 m; 72.7 ± 14.8 kg). To detect the BP in the relationship between each of FM and FFSTM and BM, a piecewise linear regression analysis was used. If a BP was detected in the corresponding relationship, the significant difference between the regression slopes above and below the BP was examined.

**Results:**

The regression analysis indicated that the BM_BP_ existed in the FM- and FFSTM-BM relationships regardless of region and whole body. For the whole body, BM_BP_ was 81.8 kg for FM and 82.2 kg for FFSTM. In regional FM-BM relationships, BM_BP_ was 80.5 kg for arms, 82.6 kg for trunk, and 63.3 kg for legs, and the regression slopes above the BM_BP_ became higher than those below the BP, and vice versa in regional FFSTM-BM relationships (BM_BP_ 104.6 kg for arms, 80.9 kg for trunk, and 79.0 kg for legs). The relative differences in the slopes between below and above BM_BP_ in the regional FM-BM relationships were higher in the arms and trunk than in the legs, and those in the regional FFSTM-BM relationships in the legs than in the trunk.

**Conclusion:**

Whole-body and regional FM- and FFSTM-BM relationships for male athletes have breakpoints at which the proportion of the tissue masses to BM alters. The BM_BP_ and differences in the distribution of regional FM and FFSTM around the breakpoint are region specific.

## Introduction

Body mass (BM) mainly consists of fat (FM), bone, and fat-free soft tissue (FFSTM) masses. FFSTM has been shown to strongly associate with whole-body and appendicular skeletal muscle masses [1]. Some earlier findings have suggested that for athletes, an increase of FM may be a factor of increasing the musculoskeletal injury risk of lower extremity [2, 3]. On the other hand, fat-free soft tissue mass (FFM) is a potential determinant of maximal force-generating capacities [4] and one of the indicators for identifying prospective athletes [5]. Therefore, the evaluation of FM and FFSTM accumulation within a body in athletes may provide useful information for us to identify prospective athletes and to design body composition for decreasing musculoskeletal injury risk and improving force generation capability.

Whole-body FM and FFM strongly associates with body size (e.g., height and BM) in athletes and untrained individuals [6–9]. However, some studies have shown that there is a breakpoint (BP), at which the regression slope alters below and above the BP, in the FM and FFM-BM [8], and FFSTM-BM relationships [9], respectively. For example, Bosch et al. [8] observed the BP at 114 kg of BM in American football players. Furthermore, the regression slope of the FFM-BM relationship above the body mass at BP (BM_BP_) becomes lower than that below the point and vice versa in the FM-BM relationship [8]. These findings indicate that the magnitude of each of FM and FFSTM accumulation within a body differs around the BP. Elucidating the BM_BP_ in FM- and FFSTM-BM relationships deepens the knowledge concerning the degree of FM and FFSTM accumulation for a given BM.

Abe et al. [10] have demonstrated that the relationship between skeletal muscle mass and body mass is nonlinear. Furthermore, Kondo et al. [6] show that thigh muscle cross-sectional area increases with increasing FFM, but a further increase in thigh muscle size is not apparent in FFM over 80 kg. These findings indicate a possibility that there is an upper limit in skeletal muscle accumulation within a body. From the viewpoint of region-specific muscle development, Wakahara et al. [11] have revealed that the variability of limb muscle size is greater in upper limb muscles than in lower limb muscles, interpreting as muscle-related differences in hypertrophic responsiveness to daily use and/or physical training. It is known that training-induced hypertrophic change is greater in upper limb muscles than in lower limb muscles [12, 13]. Furthermore, some earlier findings have demonstrated that loss of FM with physical training is region specific, and the magnitude of the loss is greater in the arms and trunk than in legs [14, 15]. The region-specific loss of FM may be caused by regional differences in fat cell metabolism [16, 17]. Taken together, it is reasonable to assume that FM and FFSTM accumulation within a body is region specific, and consequently it will produce region-specific breaking points in either FM- or FFSTM-BM relationships. However, less information on the existence of region-specific breakpoint in FM- and FFSTM-BM relationships are available from earlier studies.

The present study aimed to elucidate the BM_BP_ in whole and regional FM- and FFSTM-BM relationships for male athletes. We hypothesized that in male athletes, the regional FM and FFSTM-BM relationships have a BP, but the body mass at BP (BM_BP_) differs among the arms, trunk, and legs.

## Methods

### Participants

A total of 198 male athletes (20.8 ± 2.1 years; 1.73 ± 0.07 m; 72.7 ± 14.8 kg) voluntarily participated in this study. The inclusion criterion for the athletes was current involvement in competitive sports at national and international levels. The investigation was conducted during in-season for all subjects. Participants consisted of kendo athletes (*N* = 12), judo athletes (*N* = 37), jumpers (*N* = 13), shot put and javelin throwers (*N* = 14), gymnasts (*N* = 16), cyclists (*N* = 21), middle- and long-distance runners (*N* = 27), rugby players (*N* = 10), and soccer players (*N* = 48). They had participated in regular event-specific training for more than five days (> 1.5 h/day) per week for at least 5 years. They were free of cardiovascular, metabolic, and immunologic disorders and/or orthopedic abnormalities and were not using any medications that affected their muscular function and size. This investigation was conducted according to the Declaration of Helsinki and was approved by the local Ethics Committee for human experimentation. Prior to the experiment, all participants were informed of the experimental procedures of this study and the possible risks of the measurements beforehand. Written informed consent was obtained from each participant.

### Measurements of anthropometry

Height and body mass were measured using a stadiometer and a leg-to-leg bioelectrical impedance analyzer with a computer-programmed athletic mode (DC-320, TANITA, Japan) to the nearest 0.1 cm and 0.1 kg, respectively. Participants were instructed to restrain from alcohol intake for 24 h prior to the experiment and from having a meal 2 h prior to the measurement.

### Measurements of body composition

Percent fat mass (%FM), whole body, and regional body composition were estimated using a whole-body dual-energy X-ray absorptiometry (DXA) scanner (Hologic Delphi A-QDR, USA). Participants lay supine on a bed with arms and legs straight. Room temperature was usually kept at 22 °C. DXA-derived body composition has been shown to have good accuracy and reliability in team sport athletes [18]. To confirm the reproducibility of the DXA measurement, we measured body composition twice at least 3 days apart for nine male athletes. The intra-class correlation coefficients were 0.92 for FM and 0.91 for FFSTM. The measurement errors between 1st and 2nd measurements were − 0.6 ± 0.6 kg for FM and 0.3 ± 2.0 kg for FFSTM and the coefficient of variance were 5.4 ± 4.5% for FM and 1.8 ± 1.4% for FFSTM, respectively.

From the obtained radiography, we divided the body into four segments: the head, trunk, arms, and legs with built-in software (Hologic Delphi A-QDR, USA) according to the earlier study [19]. The arms were separated from the trunk by localizing a cut through the axilla and to the medial head of the humerus. The legs were separated from the trunk by positioning an angle cut through the bottom of the ischium, forming a triangle with the supracrestal line. The head was separated from the trunk by cutting just below the mandible. The independent variables were whole-body and regional FM, FFSTM, and bone mineral content (BMC). Trunk FFSTM was considered as the sum of the trunk skeletal muscle mass and organ-tissue mass. In addition, we calculated whole-body FFM by adding BMC to FFSTM to discuss the upper limit of FFM accumulation for a given BM in Japanese male athletes. After adjusting technical error (1.8 kg) for estimating FFM, underwater weighing method vs. DXA method [20], the regression equations of bodybuilders, weightlifters, and wrestlers reported in the earlier studies [6, 7] were added to the FFM-BM relationship obtained in this study. To exclude the impact of body size [21], whole-body FM, FFSTM, and FFM were divided into height squared (fat mass index, FMI; fat-free soft tissue mass index, FFSTMI; fat-free mass index, FFMI), respectively.

### Statistical analysis

Descriptive data are presented as mean ± SD. A piecewise linear regression analysis was used to identify the BP in each of the whole-body and regional FM-, FFSTM-, and FFM-BM relationships; whole-body FMI-, FFSTMI-, and FFMI-BM relationships; and whole-body FMI-FM, FFSTMI-FFSTM, and FFMI-FFM relationships, respectively. As described in the earlier work [22], BP was defined as the minimal residual sum of squares of two regression lines in the corresponding relationships. We tested the differences in the regression lines above and below BPs, if the regression analysis detected a BP. The difference in regression lines was also tested for FFSTM-BM vs. FFM-BM relationships, FFSTMI-BM vs. FFMI-BM relationships, and FFSTMI-FFSTM vs. FFMI-FFM relationships. The probability level for all statistical analysis was set at *p* < 0.05. All statistical analyses were conducted using a statistical software program (SPSS statistics 25.0, IBM Co., New York, USA).

## Results

**Fig. 1:**
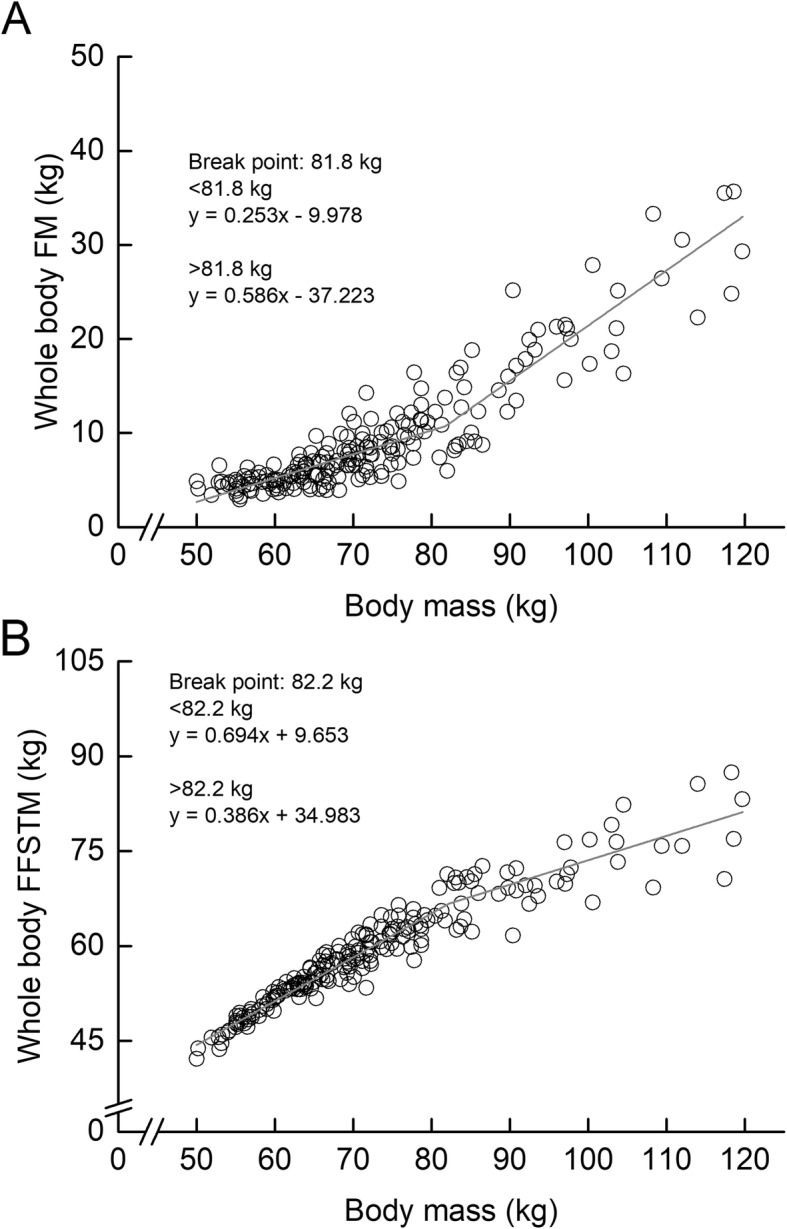
Relationships between body mass and each of whole-body fat mass (FM) (**a**) and fat-free soft tissue mass (FFSTM) (**b**). Grey solid line represents the regression line of the corresponding relationships

**Fig. 2:**
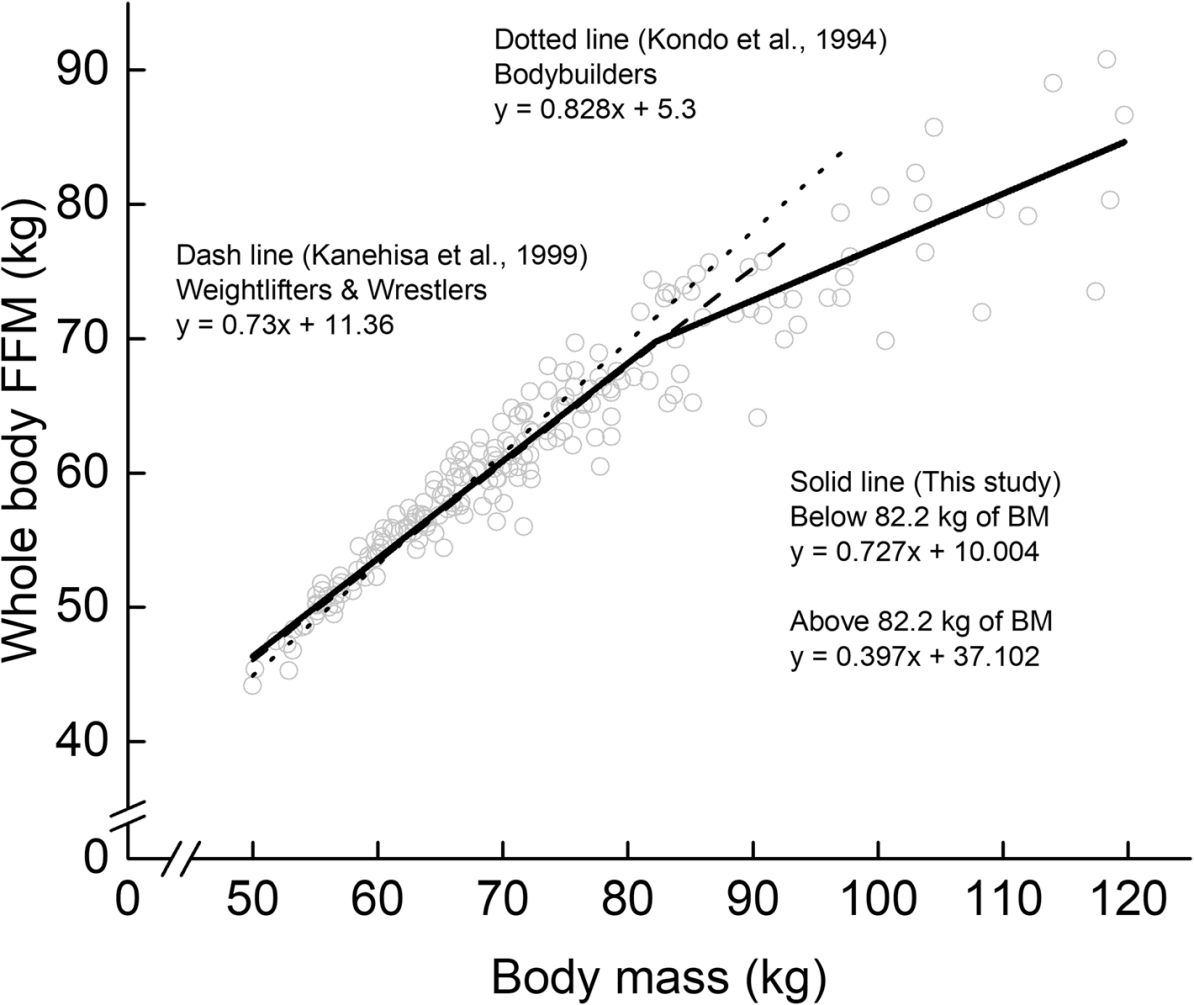
Association of whole-body fat-free mass (FFM) with body mass in Japanese male athletes. Grey open circles represent individual data of FFM obtained from this study

Descriptive data on the measured variables are shown in Table 1. The piecewise linear regression analysis revealed that whole-body FM- and FFSTM-BM relationships had the breakpoints (Fig. 1). The BM_BP_s in the FM- and FFSTM-BM relationships were 81.8 kg and 82.2 kg, respectively. In the whole-body FM-BM relationship, the regression slope above the BM_BP_ (0.59) was significantly higher than that below the BM_BP_ (0.25). In the whole-body FFSTM-BM relationship, the regression slope above the BM_BP_ (0.39) was significantly lower than that below the BM_BP_ (0.69). The whole-body FFM-BM relationship also had a BP, corresponding to 82.2 kg of BM (Fig. 2). Above the BM_BP_, the slope obtained in this study (0.40) was lower than that examined for bodybuilders and weightlifters in earlier studies (0.73–0.83).

**Table 1:** Descriptive data on body composition in male athletes

	Means		SDs	Min	Max
Height, m	1.73	±	0.07	1.57	1.88
Body mass, kg	72.7	±	14.8	50.0	119.7
BMI, kg/m^2^	24.2	±	4.1	18.4	39.5
FM, kg					
Whole body	9.4	±	6.4	2.9	35.7
Arms	0.9	±	0.7	0.2	4.0
Trunk	4.5	±	3.7	1.2	19.9
Legs	3.1	±	2.1	0.6	12.1
Head	0.9	±	0.1	0.6	1.3
%FM,%	12.3	±	5.5	5.4	32.6
FMI, kg/m^2^	3.1	±	2.0	1.1	11.4
FFSTM, kg					
Whole body	59.2	±	8.6	42.1	87.5
Arms	6.6	±	1.5	3.8	11.0
Trunk	28.8	±	4.4	21.0	48.0
Legs	20.2	±	3.0	14.6	29.7
Head	3.6	±	0.3	2.8	4.8
%FFSTM, %	82.3	±	5.3	60.1	89.1
FFSTMI, kg/m^2^	19.7	±	2.2	15.7	29.2
Bone mineral content, kg					
Whole body	2.7	±	0.4	1.6	3.8
Arms	0.4	±	0.1	0.2	0.6
Trunk	0.8	±	0.2	0.4	1.3
Legs	1.0	±	0.2	0.6	1.4
Head	0.5	±	0.1	0.3	0.8
FFM, kg					
Whole body	61.9		9.0	44.2	90.8
Arms	7.0		1.6	3.9	11.6
Trunk	29.6		4.5	21.2	48.6
Legs	21.2		3.1	15.3	31.0
Head	4.1		0.4	3.1	5.4
FFMI, kg/m^2^	20.6		2.3	16.4	30.3

*BMI* body mass index, *FM* fat mass, *%FM* percentage of fat mass in body mass, *FMI* fat mass index, *FFSTM* fat-free soft tissue mass, *%FFSTM* percentage of fat-free soft tissue mass in body mass, *FFSTM* fat-free soft tissue mass index, *FFM* fat-free mass, *FFMI* fat-free mass index

**Fig. 3:**
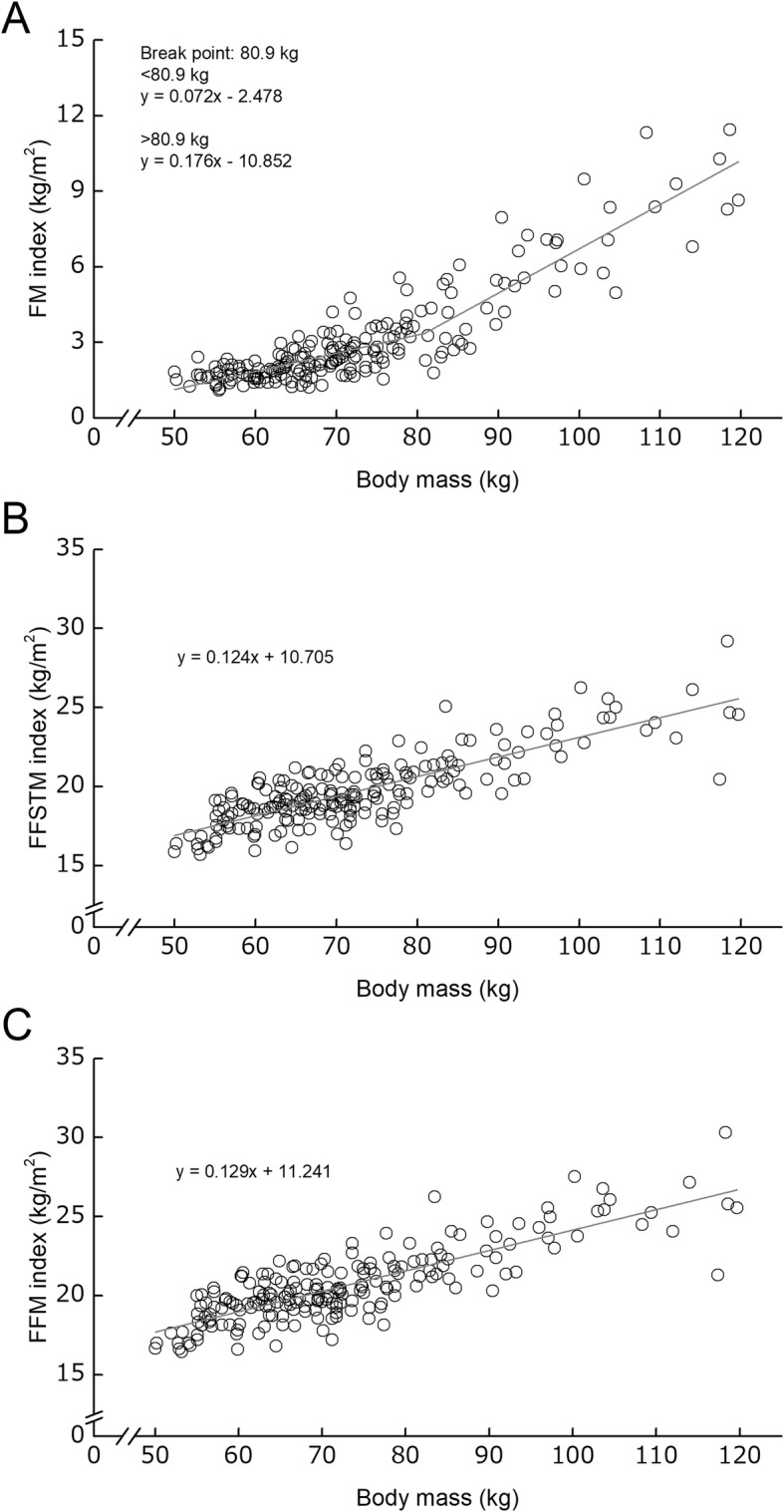
Relationships between body mass and each of whole-body fat mass index (FM index) (**a**), fat-free soft tissue mass index (FFSTM index) (**b**), and fat-free mass index (FFM index). Grey solid line represents the regression line of the corresponding relationships

The regression analysis indicated that whole-body FMI-BM relationship had a BP, corresponding to 80.9 kg of BM (Fig. 3). On the other hand, there was no BP in whole-body FFSTMI- and FFMI-BM relationships (Fig. 3).

**Fig. 4:**
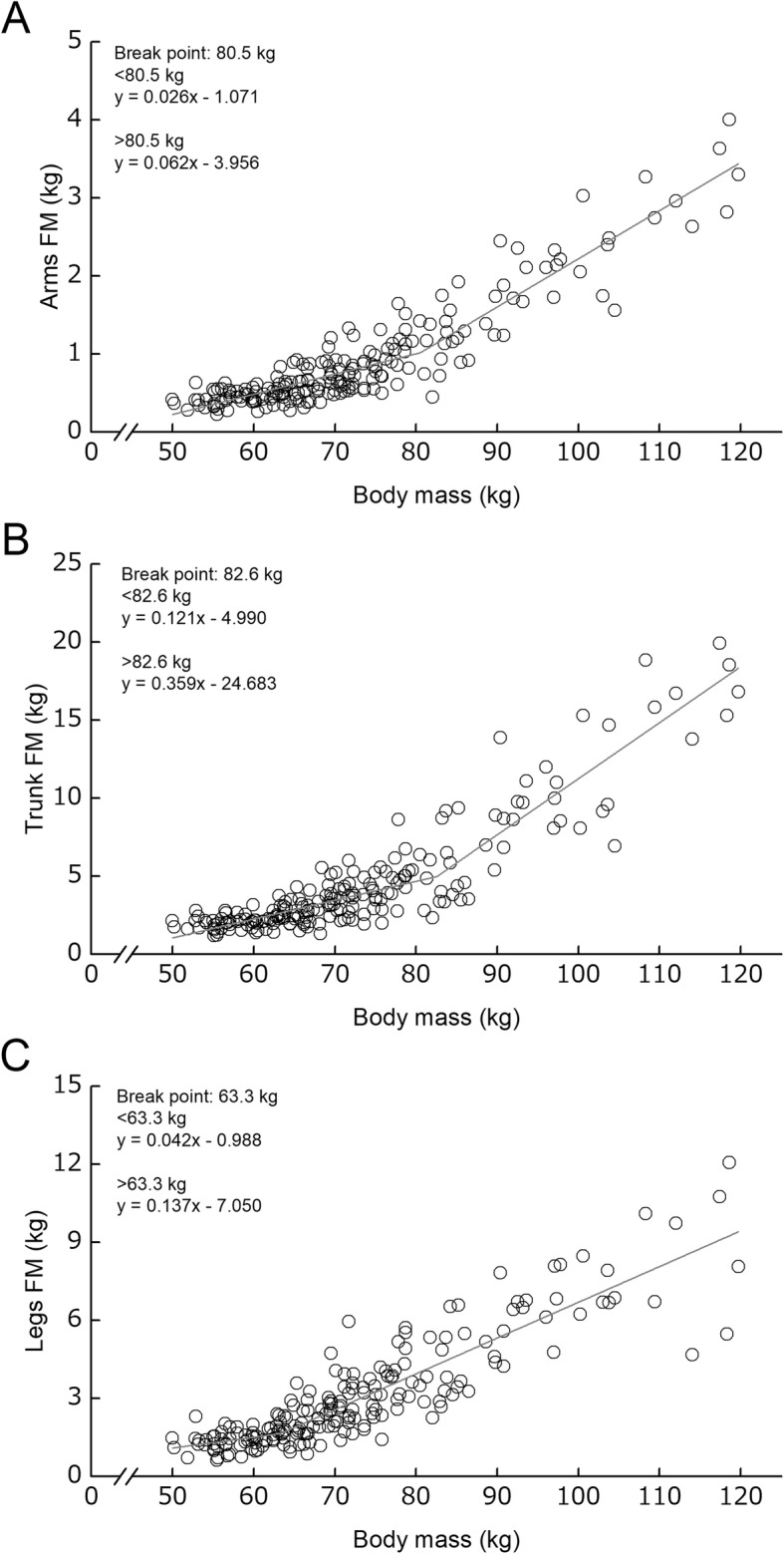
Relationships between body mass and regional fat mass (FM) in each of the arms (**a**), trunk (**b**), and legs (**c**). Grey solid line represents the regression line of the corresponding relationships

Regional FM-BM relationships had BPs regardless of segments (Fig. 4). The BM_BP_ was 80.5 kg for arms, 82.6 kg for trunk, and 63.3 kg for legs. In all segments, the regression slopes above the BM_BP_ were significantly higher than those below the BM_BP_. The regression slopes above the BM_BP_ were higher than those below the BM_BP_. The ratio in the regression slope between below and above the BM_BP_ was 2.38 for arms, 2.97 for trunk, and 3.26 for legs, respectively.

**Fig. 5:**
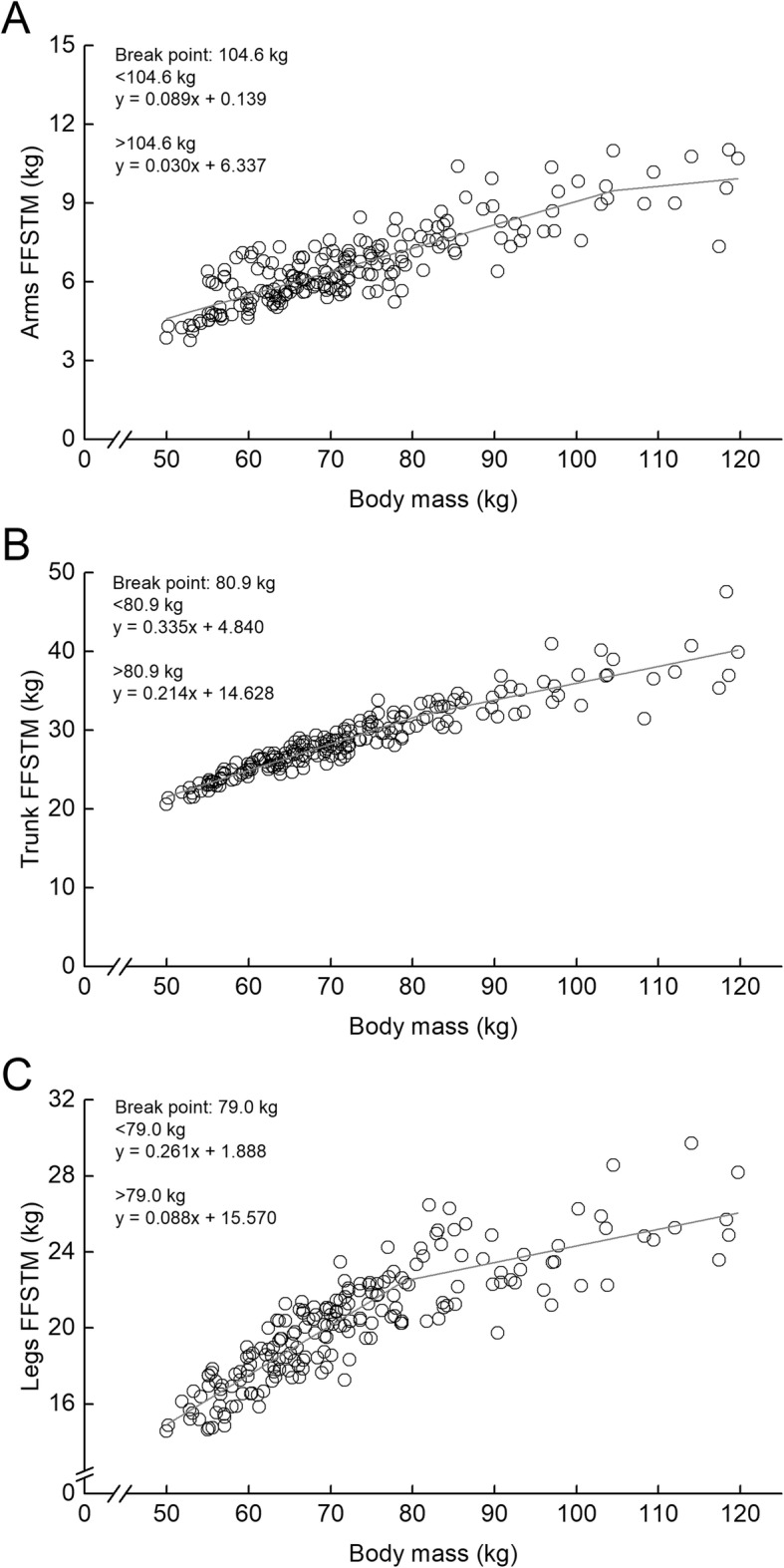
Relationships between body mass and regional fat-free soft tissue mass (FFSTM) in each of arms (**a**), trunk (**b**), and legs (**c**). Grey solid line represents the regression line of the corresponding relationships

Regional FFSTM-BM relationships had BPs in all segments (Fig. 5). The BM_BP_ was 104.6 kg for arms, 80.9 kg for trunk, and 79.0 kg for legs, respectively. In each segment, the regression slope above the BM_BP_ was significantly lower than that below the BM_BP_. The ratio in the regression slope between below- and above BM_BP_ was 0.34 for arms, 0.64 for trunk, and 0.34 for legs, respectively.

**Fig. 6:**
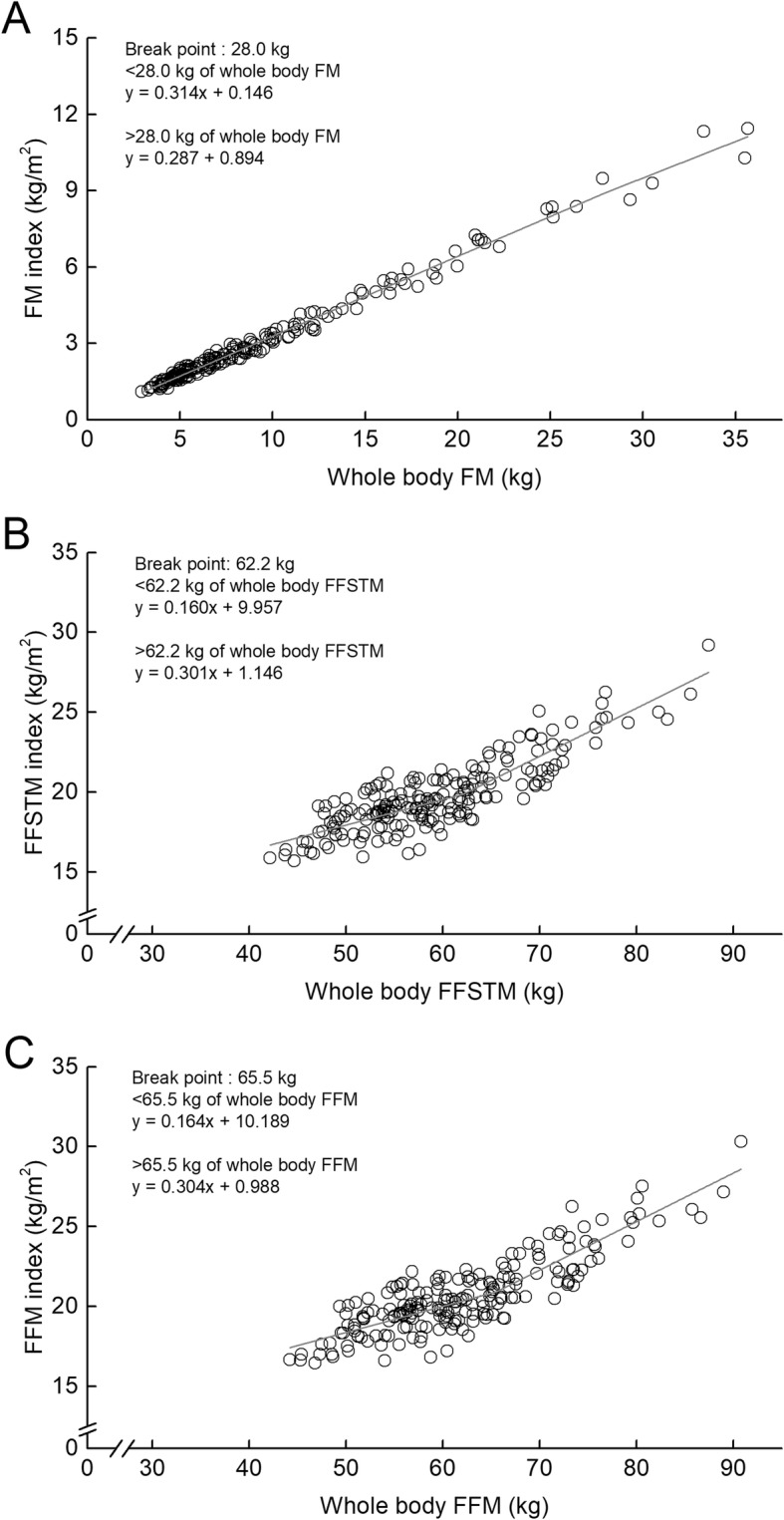
Relationships between fat mass index (FM index) and whole-body fat mass (**a**), between fat-free soft tissue mass index (FFSTM index) and whole-body fat-free soft tissue mass (**b**), and between fat-free mass index (FFM index) and whole-body fat-free mass (**c**). Grey solid line represents the regression line of the corresponding relationships

Figure 6 presents whole-body FMI-FM, FFSTMI-FFSTM, and FFMI-FFM relationships. The piecewise regression analysis revealed that the corresponding relationships had BPs. The value of BP was 28.0 kg for FM, 62.2 kg for FFSTM, and 65.5 kg for FFM. In the FMI-FM relationship, the regression slope above the BP was significantly lower than that below the BP. In the FFSTMI-FFSTM and FFMI-FFM relationships; however, the slopes above the BP were significantly higher than those below the BP.

No significant differences were found between the regression slopes for the FFSTM- and FFM-BM relationships, between those for the FFSTMI- and FFMI-BM relationships, and between those for FFSTMI-FFSTM relationship and FFMI-FFM relationship.

## Discussion

As expected, the regional FM- and FFSTM-BM relationships had BPs regardless of segments, and the BM_BP_ differed among the arms, trunk, and legs in male athletes. For the FM, the BM_BP_ was greater in the trunk than in both limbs. For the FFSTM, the BM_BP_ was smaller in the legs compared to the trunk and arms. These findings indicate that the BM-related differences in regional FM and FFSTM accumulation are region specific. Furthermore, in the regional FM and FFSTM relationships, the regression slopes below and above the BM_BP_ also differed among the segments. The segment-related difference in the regression slope allows us to understand the proportion of either FM or FFSTM to BM.

The BM_BP_s of the whole-body FM- and FFSTM-BM relationships (FM, 81.8 kg of BM; FFSTM, 82.2 kg of BM) were different from those reported in the earlier findings [8, 9]. The values obtained here were considerably smaller than that reported for American footballers (114 kg of BM) [8]. However, the BM_BP_ in FFSTM-BM relationship was greater as compared to that observed in our previous study [9] which examined male athletes and untrained males (72.4 kg of BM). Distribution of FM and FFSTM within a body has been shown to be influenced by training status and ethnicity [19, 23]. Stewart et al. [23] demonstrated that the proportion of regional FM and FFSTM to BM differed between athletes and untrained individuals. Furthermore, it has been reported that the proportion of FM and FFSTM to BM in Australian rugby players differs between the Caucasian and Polynesian, whereas no significant position-related differences are found between both ethnic groups [19]. Therefore, training status- and/or ethnic-related differences in the participants examined may be factors explaining the observed differences in the BPs of FM- and FFSTM-BM relationships between the present and earlier studies.

In the whole-body FFSTM-BM relationship, the regression slopes below and above BM_BP_ (82.2 kg of BM) were 0.694 and 0.386, respectively. It indicates that the proportion of whole-body FFSTM to BM is smaller in the athletes with above BM_BP_ than those with below BM_BP_. One of the factors concerning the existence of a BP in whole-body FFSTM-BM relationship may be considered that the proportion of FM accumulation to BM alters before and after the BM_BP_. In fact, whole-body FMI-BM relationship had a BP, corresponding to 80.9 kg of BM. Whole-body FFSTMI-BM relationship had no BP, indicating that the relationship was linear. Furthermore, the FFSTMI-FFSTM relationship had a BP, corresponding to 62.2 kg of whole-body FFSTM. Substituting the value (*y*) into the regression equation (*y* = 0.694*x* + 9.653), one finds the BP corresponding to 75.7 kg of BM. This implies that in male athletes, whole-body FFSTM relative to body height squared becomes higher if BM is over 75.7 kg. Taken together, it can be considered that the existence of the BP in whole-body FFSTM-BM relationship might be due to greater proportion of FM accumulation above a given BM.

As seen in Fig. 3, on the other hand, the slope of the whole-body FFM-BM relationship is 0.73 for highly trained Japanese weightlifters and wrestlers with < 95 kg of BM [7] and 0.828 for Japanese bodybuilders with < 100 kg of BM [6]. Weight-classified athletes such as weightlifters and wrestlers are required to control their BM for adjusting to their own weight classes and to maximize skeletal muscle mass within the prescribed BM. Similarly, bodybuilders generally design their own training regimen to induce greater muscle hypertrophy. These aspects will be a background for the fact that the percentage of whole-body FFM to BM is higher in bodybuilders [6] and weightlifters and wrestlers [7] (approximately 89% of BM) than male athletes examined here (86% of BM). In addition, the breakpoint found in this study has not been shown in the FFM-BM relationships for the bodybuilders [6] and the weight-classified athletes [7]. Combining the current findings with the earlier findings, it is considered that we can present the upper limit of FFM accumulation for a given BM in Japanese male athletes (Fig. 3). In fact, the regression lines derived from the equations reported in the earlier studies overlapped with that obtained here below the BP (82.2 kg of BM). Above the BM_BP_, however, the regression slope for the athletes examined here (0.40) was lower than that reported by earlier studies (073–0.83) [6, 7]. Taken together, it may be assumed that in Japanese male athletes with less than 82.2 kg of BM, FFM is linearly associated with BM while preserving the proportion of FFM to BM to be nearly 0.8 regardless of sport events, but the ratio would fall off in athletes with over 82.2 kg of BM, being not categorized as strength-trained athletes.

In the regional FFSTM-BM relationships, the BPs were found regardless of segments, and the BM_BP_ differed among segments. The BM_BP_ was greater in arms (104.6 kg) than in trunk (80.9 kg) and legs (79.0 kg). This suggests that as compared to trunk and legs, arms can store FFSTM to a greater extent of BM without change in its proportion to BM. Site-specific difference in the relationship between individual muscle size and whole-body FFM might be involved as a physiological mechanism yielding segment-related difference in the BP in the regional FFSTM-BM relationships. Kondo et al. [6] demonstrated that as FFM increases, thigh muscle cross-sectional area (CSA) also increases until 80 kg of FFM, but a further increase in the CSA is not apparent in male athletes with FFM over 80 kg, although FFM is linearly related with body mass. This indicates that thigh muscles may not accumulate in a body beyond a given FFM. Abe et al. [10] also demonstrated that the relationship between skeletal muscle mass and whole body is nonlinear. To the best of our knowledge, no studies have examined how muscle size of segments other than thigh can be associated with either BM or FFM. If the earlier findings on the thigh muscles can be applied to other individual muscles, it would be a reason for the nonlinear relationship between regional FFSTMs and BM in this study.

In the regional FM-BM relationships, there were also region-related differences in the BM_BP_ (arms, 80.5 kg; trunk, 82.6 kg; legs, 63.3 kg). Contrary to FFSTM, the regression slopes above the BM_BP_ became steeper than those below the BM_BP_ in all segments. The slopes above the BM_BP_ were greater in arms (0.026 to 0.062) and trunk (0.121 to 0.359) than legs (0.042 to 0.137) as compared to that below the BM_BP_. Nindl et al. [24] demonstrated that male soldiers who were overweight had a greater percentage of arms FM to BM as compared to those with normal and low weight, in spite of the fact that no group differences in the proportion of trunk and legs FMs to BM are found. In addition, as a result of longitudinal observation for 1 year, adipose tissue increase at triceps site increased in deep and superficial subcutaneous layers, but at the abdominal site, the corresponding increase was found in superficial subcutaneous layer only [25]. Rognum et al. [26] revealed that prolonged exercise and severe energy deficiency brought an intracellular fat reduction in the gluteal and abdominal regions but not in the femoral site. These findings indicate that deposition and lipolytic action differ among trunk and limbs. These regional differences in the extent of FM accumulation may be due to the site-related differences in triglyceride storage capacity [27] and/or lipolysis of fat cell [28] mediated by catecholamines [26]. Taken together, it can be said that the observed region-related differences in the breakpoints of regional FM-BM relationships may be attributable to those in the susceptibility to fat tissue accumulation in the corresponding segments.

The present study has some limitations to discuss FM- and FFSTM-BM relationships in male athletes. Firstly, the maximal value of BM for the athletes examined here was 119.7 kg. This was lower than that (< 181 kg of BM) of the participants examined in the earlier studies [6, 8]. Bosch et al. [8] demonstrated that BM_BP_ of FM- and FFSTM-BM relationships is 114 kg. Therefore, there is a possibility that the BM_BP_ obtained here might alter if heavier athletes are examined. As mentioned above, however, the relationship between individual muscle size and BM or FFM is nonlinear, indicating that the ratios of thigh muscle CSA to FFM, and the ratio of skeletal muscle mass to BM may be nearly constant in spite of the magnitude of FFM and BM, respectively [6, 10]. These findings will deny the possibility that the BM_BP_ obtained here might alter if heavier athletes are examined. Secondly, there is a possibility that the BM_BP_ obtained in this study might depend on the type of the examined events. In further analysis, judo athletes and throwers showed greater FM and FFSTM, compared to runners and gymnasts (Suppl. 1). In addition, the proportion of FM to BM was higher in the heavier athletes than the lighter athletes. Thus, we cannot rule out that the BM_BP_ might be affected by the type of athletic events. Further investigation is needed to clarify this point. Thirdly, FFSTM involves not only skeletal muscle mass but also other organ-tissue mass. The regression slopes of the FFSTM-BM relationship would be potentially influenced by inter-individual differences in the other organ-tissue mass. Midorikawa et al. [29] have demonstrated that skeletal muscle, liver, and kidney masses are linearly associated with FFM in male college athletes and the regression slopes of the tissue mass-FFM relationships are 0.49 for skeletal muscle mass, 0.007 for kidney mass, and 0.04 for liver mass. This finding suggests that the whole-body and trunk FFSTM-BM relationships proposed here might involve more or less the influence of the mass of organ tissues such as the kidney and liver.

This study demonstrates that for male athletes, an increase in BM leads to gains in both FM and FFSTM. Furthermore, the FFSTMI-FFSTM relationship had a BP, corresponding to 62.2 kg of FFSTM. The rate of increase in FFSTMI was higher above the BP, compared to that below the BP. This indicates that the male athletes with FFSTM above BP may have greater FFSTM relative to body size, compared to ones with FFSTM below BP. The FM- and FMI-BM relationships also demonstrated that the rate of increase in fat tissue mass with increasing BM may be higher in male athletes with over 81 kg of BM than in those with less than 81 kg of BM. Taken together, the current findings suggest that the heavier male athletes with a BM above BP need to increase fat-free tissue mass and to decrease fat tissue mass, compared to the lighter ones with a BM below BP. In particular, the prescription may focus on legs and trunk fat-free tissue masses because of the lower proportion of fat-free tissue accumulation in the legs and trunk segment for the heavier male athletes. So the BM_BP_ obtained in this study may be useful information for male athletes and their coaches to design a weight management program, including physical exercises, for increasing FFSTM within a given BM.

## Conclusion

This study demonstrates that whole-body and regional FM- and FFSTM-BM relationships for male athletes have breakpoints at which the proportion of FM and FFSTM accumulation to BM alters. The magnitude of BM at the breakpoint and the change in the proportion around the breakpoint are region specific. On the other hand, the physiological mechanisms for the region-related difference in BM_BP_ and the plasticity of FM and FFSTM (e.g., physical training and weight reduction) for heavier male athletes with above BM_BP_ are uncertain. Further investigations are needed to enhance understanding of the plasticity of FM and FFSTM for a given BM for male athletes.

## Supplementary information

**Additional file 1.** Suppl. 1 Event-related differences in anthropometry and body composition in male athletes.

## Data Availability

The datasets during and/or analyzed during the current study are available from the corresponding author on reasonable request.

## Abbreviations

BM: Body mass
BM_BP_: Body mass at the breakpoint
BP: Breakpoint
CSA: Cross-sectional area
FFM: Fat-free mass
FFMI: Fat-free mass index
FFSTM: Fat-free soft tissue mass
FFSTMI: Fat-free soft tissue mass index
FM: Fat mass
FMI: Fat mass index

## References

[1] Kim J, Wang Z, Heymsfield SB, Baumgartner RN, Gallagher D (2002). Total-body skeletal muscle mass: estimation by a new dual-energy X-ray absorptiometry method. Am J Clin Nutr..

[2] Kaplan TA, Digel SL, Scavo VA, Arellana SB (1995). Effect of obesity on injury risk in high school football players. Clin J Sport Med..

[3] Nye NS (2014). Abdominal circumference is superior to body mass index in estimating musculoskeletal injury risk. Med Sci Sports Exerc..

[4] Brechue WF, Abe T (2002). The role of FFM accumulation and skeletal muscle architecture in powerlifting performance. Eur J Appl Physiol..

[5] Takai Y (2017). Lean body mass index is an indicator of body composition for screening prospective young adult soccer players. Football Sci..

[6] Kondo M, Abe T, Ikegawa S, Kawakami Y, Fukunaga T (1994). Upper limit of fat-free mass in humans: A study on Japanese Sumo wrestlers. Am J Hum Biol..

[7] Kanehisa H, Ikegawa S, Fukunaga T (1998). Body composition and cross-sectional areas of limb lean tissues in Olympic weight lifters. Scand J Med Sci Sports..

[8] Bosch TA (2014). Abdominal body composition differences in NFL football players. J Strength Cond Res..

[9] Takai Y (2018). Body shape indices are predictors for estimating fat-free mass in male athletes. PloS One..

[10] Abe T (2018). Skeletal muscle mass in human athletes: What is the upper limit?. Am J Hum Biol..

[11] Wakahara T (2010). Variability of limb muscle size in young men. Am J Hum Biol..

[12] Abe T, DeHoyos DV, Pollock ML, Garzarella L (2000). Time course for strength and muscle thickness changes following upper and lower body resistance training in men and women. Eur J Appl Physiol..

[13] Wernbom M, Augustsson J, Thomee R (2007). The influence of frequency, intensity, volume and mode of strength training on whole muscle cross-sectional area in humans. Sports Med..

[14] Nindl BC (2000). Regional body composition changes in women after 6 months of periodized physical training. J Appl Physiol (1985).

[15] Ramirez-Campillo R (2013). Regional fat changes induced by localized muscle endurance resistance training. J Strength Cond Res..

[16] Smith U, Hammersten J, Bjorntorp P, Kral JG (1979). Regional differences and effect of weight reduction on human fat cell metabolism. Eur J Clin Invest..

[17] Jensen MD (1995). Gender differences in regional fatty acid metabolism before and after meal ingestion. J Clin Invest..

[18] Bilsborough JC (2014). The accuracy and precision of DXA for assessing body composition in team sport athletes. J Sports Sci..

[19] Zemski AJ, Slater GJ, Broad EM (2015). Body composition characteristics of elite Australian rugby union athletes according to playing position and ethnicity. J Sports Sci..

[20] Wellens R (1994). Body composition in white adults by dual-energy x-ray absorptiometry, densitometry, and total body water. Am J Clin Nutr..

[21] VanItallie TB, Yang MU, Heymsfield SB, Funk RC, Boileau RA (1990). Height-normalized indices of the body's fat-free mass and fat mass: potentially useful indicators of nutritional status. Am J Clin Nutr..

[22] Vieth E (1989). Fitting piecewise linear regression functions to biological responses. J Appl Physiol (1985).

[23] Stewart AD, Hannan J (2000). Sub-regional tissue morphometry in male athletes and controls using dual X-Ray absorptiometry (DXA). Int J Sport Nutr Exerc Metab..

[24] Nindl BC (1996). Regional fat placement in physically fit males and changes with weight loss. Med Sci Sports Exerc..

[25] Alexander HG, Dugdale AE (1992). Fascial planes within subcutaneous fat in humans. Eur J Clin Nutr..

[26] Rodahl K, Opstad PK, Rognum TO (1982). Regional differences in the lipolytic response of the subcutaneous fat depots to prolonged exercise and severe energy deficiency. Eur J Appl Physiol Occup Physiol..

[27] Rebuffe-Scrive M (1985). Fat cell metabolism in different regions in women. Effect of menstrual cycle, pregnancy, and lactation. J Clin Invest..

[28] Wahrenberg H, Lonnqvist F, Arner P (1989). Mechanisms underlying regional differences in lipolysis in human adipose tissue. J Clin Invest..

[29] Midorikawa T, Sekiguchi O, Beekley MD, Bemben MG, Abe T (2007). A comparison of organ-tissue level body composition between college-age male athletes and nonathletes. Int J Sports Med..

