# Supplementary material for: Profile of regional fat and fat-free soft tissue accumulation in male athletes
Source: J Physiol Anthropol. 2020 Mar 6;39:5. doi: 10.1186/s40101-020-0215-0 (PMC7059667; doi:10.1186/s40101-020-0215-0)
Supplement: Supplementary file 1 — Additional file 1. Suppl. 1 Event-related differences in anthropometry and body composition in male athletes. [file 40101_2020_215_MOESM1_ESM.docx]

Suppl. 1 Event-related differences in anthropometry and body composition in male athletes.

|  | **Runner**  **(27)** | | | **Gymnast**  **(16)** | | | **Jumper**  **(13)** | | | **Cyclist**  **(21)** | | | **Kendo**  **(12)** | | | **Soccer**  **(48)** | | | **Rugby**  **(10)** | | | **Judo**  **(37)** | | | **Thrower**  **(14)** | | | **Post hoc test** |
| --- | --- | --- | --- | --- | --- | --- | --- | --- | --- | --- | --- | --- | --- | --- | --- | --- | --- | --- | --- | --- | --- | --- | --- | --- | --- | --- | --- | --- |
| **Height, m** | 1.70 | ± | 0.06 | 1.63 | ± | 0.04 | 1.74 | ± | 0.05 | 1.73 | ± | 0.06 | 1.71 | ± | 0.04 | 1.76 | ± | 0.07 | 1.75 | ± | 0.06 | 1.73 | ± | 0.06 | 1.77 | ± | 0.04 | **GYM<Others; RUN<S,TRW** |
| **Body mass, kg** | 57.4 | ± | 5.3 | 58.9 | ± | 2.8 | 67.3 | ± | 4.9 | 69.3 | ± | 7.9 | 71.1 | ± | 7.3 | 71.4 | ± | 6.9 | 78.2 | ± | 11.3 | 88.2 | ± | 16.8 | 89.4 | ± | 16.3 | **GYM,RUN<Others**  **RUN,JMP,CYC,KND,S<JD,TRW** |
| **BMI, kg/m^2^** | 19.9 | ± | 1.1 | 22.2 | ± | 1.0 | 22.1 | ± | 1.3 | 23.1 | ± | 1.7 | 24.2 | ± | 2.2 | 22.9 | ± | 1.4 | 25.4 | ± | 2.4 | 29.2 | ± | 4.5 | 28.5 | ± | 5.0 | **RUN<Others**  **S,JMP,GYM,CYC <JD,TRW** |
| **FM, kg** |  |  |  |  |  |  |  |  |  |  |  |  |  |  |  |  |  |  |  |  |  |  |  |  |  |  |  |  |
| **Whole body** | 4.8 | ± | 1.2 | 4.9 | ± | 0.8 | 5.6 | ± | 1.2 | 7.3 | ± | 1.9 | 10.4 | ± | 3.3 | 7.8 | ± | 2.7 | 12.3 | ± | 3.7 | 15.3 | ± | 9.0 | 17.6 | ± | 8.2 | **JMP,GYM,RUN<S,KND,RGB,JD,TRW**  **GYM,RUN<CYC,S** |
| **Arms** | 0.4 | ± | 0.1 | 0.5 | ± | 0.1 | 0.5 | ± | 0.1 | 0.7 | ± | 0.2 | 1.0 | ± | 0.4 | 0.7 | ± | 0.3 | 1.2 | ± | 0.3 | 1.6 | ± | 1.0 | 1.8 | ± | 0.9 | **JMP,GYM,RUN<S,KND,RGB,JD,TRW**  **RUN<CYC,S; GYM<S** |
| **Trunk** | 2.1 | ± | 0.6 | 2.0 | ± | 0.3 | 2.5 | ± | 0.7 | 3.3 | ± | 1.0 | 4.8 | ± | 1.8 | 3.5 | ± | 1.3 | 6.0 | ± | 2.2 | 7.6 | ± | 5.4 | 9.2 | ± | 5.0 | **JMP,GYM,RUN<S,KND,RGB,JD,TRW**  **GYM,RUN<CYC,S** |
| **Legs** | 1.4 | ± | 0.6 | 1.5 | ± | 0.5 | 1.8 | ± | 0.4 | 2.3 | ± | 0.7 | 3.5 | ± | 1.3 | 2.7 | ± | 1.1 | 4.2 | ± | 1.4 | 5.1 | ± | 2.8 | 5.6 | ± | 2.4 | **JMP,GYM,RUN<S,KND,RGB,JD,TRW**  **GYM,RUN<CYC,S** |
| **Head** | 0.9 | ± | 0.1 | 0.9 | ± | 0.1 | 0.9 | ± | 0.1 | 0.9 | ± | 0.1 | 1.0 | ± | 0.1 | 0.9 | ± | 0.1 | 1.0 | ± | 0.1 | 1.0 | ± | 0.1 | 1.0 | ± | 0.1 | **RUN<S,KND,CYC,JD,TRW**  **S,JMP,GYM<JD** |
| **%FM, %** | 8.4 | ± | 1.8 | 8.4 | ± | 1.2 | 8.5 | ± | 1.4 | 10.6 | ± | 2.0 | 14.6 | ± | 3.5 | 11.0 | ± | 3.1 | 15.8 | ± | 2.9 | 16.5 | ± | 7.2 | 19.3 | ± | 6.9 | **RUN<S,KND,CYC,RGB,JD,TRW**  **JUM,GYM<S,CYC<RGB,JD,TRW** |
| **FMI, kg/m^2^** | 1.6 | ± | 0.4 | 1.8 | ± | 0.3 | 1.9 | ± | 0.4 | 2.4 | ± | 0.6 | 3.5 | ± | 1.1 | 2.5 | ± | 0.8 | 4.0 | ± | 1.0 | 5.0 | ± | 2.8 | 5.6 | ± | 2.5 | **JMP,GYM,RUN<S,KND,RGB,JD,TRW**  **GYM,RUN<CYC**  **S,CYC<JD,RGB,TRW** |

**Suppl. 1**

| **FFSTM, kg** |  |  |  |  |  |  |  |  |  |  |  |  |  |  |  |  |  |  |  |  |  |  |  |  |  |  |  |  |
| --- | --- | --- | --- | --- | --- | --- | --- | --- | --- | --- | --- | --- | --- | --- | --- | --- | --- | --- | --- | --- | --- | --- | --- | --- | --- | --- | --- | --- |
| **Whole body** | 49.4 | ± | 4.5 | 50.8 | ± | 2.4 | 58.1 | ± | 4.2 | 58.3 | ± | 6.3 | 56.9 | ± | 4.7 | 59.4 | ± | 5.0 | 61.8 | ± | 7.6 | 67.9 | ± | 8.4 | 66.9 | ± | 9.0 | **GYM,RUN<S,KND,JMP,RGB,JD,TRW**  **S,KND,JMP,CYC,RUN<JD** |
| **Arms** | 4.8 | ± | 0.6 | 6.6 | ± | 0.5 | 6.4 | ± | 0.6 | 6.1 | ± | 0.9 | 6.8 | ± | 0.6 | 6.0 | ± | 0.7 | 7.0 | ± | 1.2 | 8.6 | ± | 1.4 | 7.4 | ± | 1.1 | **RUN<S,KND,JMP,GYM,CYC,RUN<JD**  **S<GYM,TRW** |
| **Trunk** | 23.9 | ± | 2.1 | 24.8 | ± | 1.2 | 27.9 | ± | 2.3 | 28.2 | ± | 2.9 | 27.4 | ± | 2.4 | 28.7 | ± | 2.6 | 30.8 | ± | 4.2 | 32.8 | ± | 4.2 | 34.0 | ± | 5.4 | **KND,JMP<JD,TRW**  **GYM,RUN<S,KND,JMP,CYC,RGB,JD,TRW** |
| **Legs** | 17.4 | ± | 1.8 | 15.9 | ± | 1.0 | 20.4 | ± | 1.6 | 20.5 | ± | 2.6 | 19.2 | ± | 1.8 | 21.1 | ± | 2.0 | 20.2 | ± | 2.2 | 22.5 | ± | 3.2 | 21.8 | ± | 2.4 | **GYM<S,KND,JMP,CYC,RGB,JD,TRW**  **RUN<S,JMP,CYC,JD,TRW** |
| **Head** | 3.4 | ± | 0.2 | 3.4 | ± | 0.2 | 3.5 | ± | 0.2 | 3.6 | ± | 0.3 | 3.7 | ± | 0.2 | 3.6 | ± | 0.2 | 3.8 | ± | 0.3 | 3.9 | ± | 0.3 | 3.9 | ± | 0.4 | **RUN<S,KND,RGB,JD,TRW**  **S,JMP,GYM<JD GYM<KND,RGB,TRW** |
| **%FFSTM, %** | 86.2 | ± | 1.8 | 86.2 | ± | 1.1 | 86.4 | ± | 1.5 | 84.2 | ± | 1.9 | 80.3 | ± | 3.2 | 83.3 | ± | 3.0 | 79.2 | ± | 2.6 | 78.1 | ± | 7.0 | 75.7 | ± | 6.9 | **S,KND,CYC,RGB,JD,TRW<GYM,RUN**  **JD,TRW<S,CYC<JMP** |
| **FFSTMI, kg/m^2^** | 17.1 | ± | 1.0 | 19.1 | ± | 0.8 | 19.1 | ± | 1.1 | 19.4 | ± | 1.2 | 19.4 | ± | 1.6 | 19.1 | ± | 1.2 | 20.1 | ± | 1.5 | 22.5 | ± | 2.0 | 21.4 | ± | 3.0 | **S,KND,JMP,GYM,CYC,RGB<JD**  **RUN<Others** |

**Suppl. 1**

| **FFM, kg** |  |  |  |  |  |  |  |  |  |  |  |  |  |  |  |  |  |  |  |  |  |  |  |  |  |  |  |  |
| --- | --- | --- | --- | --- | --- | --- | --- | --- | --- | --- | --- | --- | --- | --- | --- | --- | --- | --- | --- | --- | --- | --- | --- | --- | --- | --- | --- | --- |
| **Whole body** | 51.6 | ± | 4.7 | 53.3 | ± | 2.6 | 60.8 | ± | 4.3 | 60.7 | ± | 6.6 | 59.5 | ± | 4.8 | 62.2 | ± | 5.2 | 64.7 | ± | 8.0 | 71.0 | ± | 8.7 | 69.8 | ± | 9.2 | **GYM,RUN<S,KND,JMP,CYC,RGB,JD,TRW**  **S,KND,JMP,CYC<JD** |
| **Arms** | 5.0 | ± | 0.7 | 7.0 | ± | 0.5 | 6.7 | ± | 0.7 | 6.5 | ± | 1.0 | 7.1 | ± | 0.6 | 6.3 | ± | 0.7 | 7.4 | ± | 1.2 | 9.1 | ± | 1.4 | 7.7 | ± | 1.1 | **RUN<Others; S<KND,GYM**  **S,KND,GYM,JMP,CYC,TRW<JD** |
| **Trunk** | 24.5 | ± | 2.1 | 25.6 | ± | 1.3 | 28.7 | ± | 2.1 | 28.8 | ± | 2.9 | 28.1 | ± | 2.5 | 29.6 | ± | 2.6 | 31.6 | ± | 4.3 | 33.8 | ± | 4.2 | 34.8 | ± | 5.5 | **GYM,RUN<S,JMP,CYC,JD,TRW**  **KND,JMP,CYC<JD,TRW** |
| **Legs** | 18.2 | ± | 1.9 | 16.8 | ± | 1.0 | 21.4 | ± | 1.7 | 21.3 | ± | 2.6 | 20.1 | ± | 1.8 | 22.2 | ± | 2.1 | 21.3 | ± | 2.3 | 23.7 | ± | 3.3 | 22.9 | ± | 2.5 | **GYM<S,KND,JMP,CYC,RGB,JD,TRW**  **RUN<S,JMP,CYC,JD,RGB** |
| **Head** | 3.8 | ± | 0.3 | 3.8 | ± | 0.2 | 4.0 | ± | 0.2 | 4.1 | ± | 0.4 | 4.2 | ± | 0.2 | 4.1 | ± | 0.3 | 4.4 | ± | 0.3 | 4.4 | ± | 0.4 | 4.4 | ± | 0.4 | **GYM<KND,RGB,JD,TRW; S,JMP<JD**  **RUN<S,KND,RGB,JD,TRW** |
| **%FFM, %** | 90.0 | ± | 1.8 | 90.4 | ± | 1.2 | 90.4 | ± | 1.5 | 87.7 | ± | 2.0 | 83.9 | ± | 3.4 | 87.2 | ± | 3.1 | 83.0 | ± | 2.8 | 81.7 | ± | 7.3 | 79.0 | ± | 7.3 | **JD,TRW<S,JMP,GYM,CYC,RUN**  **S,KND,CYC,RGB,JD<GYM,RUN**  **S<JMP,GYM,RUN** |
| **FFMI, kg/m^2^** | 17.9 | ± | 1.0 | 20.0 | ± | 0.8 | 20.0 | ± | 1.1 | 20.2 | ± | 1.2 | 20.3 | ± | 1.6 | 20.0 | ± | 1.2 | 21.1 | ± | 1.6 | 23.6 | ± | 2.0 | 22.3 | ± | 3.1 | **RUN<Others**  **S,KND,JMP,GYM,CYC,RGB<JD** |

**Suppl. 1**

| **BMC, kg** |  |  |  |  |  |  |  |  |  |  |  |  |  |  |  |  |  |  |  |  |  |  |  |  |  |  |  |  |
| --- | --- | --- | --- | --- | --- | --- | --- | --- | --- | --- | --- | --- | --- | --- | --- | --- | --- | --- | --- | --- | --- | --- | --- | --- | --- | --- | --- | --- |
| **Whole body** | 2.1 | ± | 0.3 | 2.5 | ± | 0.2 | 2.7 | ± | 0.2 | 2.4 | ± | 0.3 | 2.6 | ± | 0.1 | 2.8 | ± | 0.3 | 3.0 | ± | 0.4 | 3.1 | ± | 0.4 | 2.9 | ± | 0.3 | **RUN<S,KND,JMP,GYM,RGB,JD,TRW**  **KND,JMP,GYM,CYC<S<JD**  **CYC<S,JMP,TRW,RGB**  **KND,GYM,CYC<TRHW** |
| **Arms** | 0.3 | ± | 0.1 | 0.4 | ± | 0.0 | 0.3 | ± | 0.1 | 0.3 | ± | 0.1 | 0.4 | ± | 0.0 | 0.3 | ± | 0.0 | 0.4 | ± | 0.1 | 0.5 | ± | 0.1 | 0.4 | ± | 0.0 | **RUN<Others; S,JMP<GYM**  **S,KND,JMP,TRW,GYM,CYC<JD**  **CYC<KND,TRW,GYM; S<GYM,TRW** |
| **Trunk** | 0.6 | ± | 0.1 | 0.7 | ± | 0.1 | 0.8 | ± | 0.1 | 0.7 | ± | 0.1 | 0.7 | ± | 0.1 | 0.8 | ± | 0.1 | 0.9 | ± | 0.2 | 1.0 | ± | 0.1 | 0.9 | ± | 0.1 | **RUN<Others, CYC<S,JMP,TRW**  **S,KND,JMP,CYC,GYM<JD**  **KND,GYM<S** |
| **Legs** | 0.9 | ± | 0.1 | 0.8 | ± | 0.1 | 1.1 | ± | 0.1 | 0.9 | ± | 0.1 | 0.9 | ± | 0.1 | 1.1 | ± | 0.1 | 1.1 | ± | 0.2 | 1.1 | ± | 0.1 | 1.1 | ± | 0.1 | **RUN, CYC,GYM <S,JMP,RGB,JD,TRW**  **KND<S,RGB,JD,TRW** |
| **Head** | 0.5 | ± | 0.1 | 0.5 | ± | 0.1 | 0.5 | ± | 0.0 | 0.5 | ± | 0.1 | 0.5 | ± | 0.1 | 0.5 | ± | 0.1 | 0.5 | ± | 0.1 | 0.5 | ± | 0.1 | 0.5 | ± | 0.1 |  |

BMI, body mass index; FM, fat mass; %FM, percentage of fat mass in body mass; FMI, fat mass index, FFSTM, fat-free soft tissue mass; %FFSTM, percentage of fat-free soft tissue mass in body mass; FFSTM, fat-free soft tissue mass index; FFM, fat-free mass; FFMI, fat-free mass index

RUN, runner; GYM, gymnast; JMP, jumper; CYC, cyclist; KND, kendo athlete; S, soccer athlete; JD, judo athlete; TRW, thrower
